# Association Between Insulin Resistance and Oxidative Stress Parameters in Obese Adolescents with Non-Alcoholic Fatty Liver Disease

**DOI:** 10.4274/Jcrpe.825

**Published:** 2013-03-21

**Authors:** Özgür Pirgon, Hüseyin Bilgin, Ferhat Çekmez, Hüseyin Kurku, Bumin Nuri Dündar

**Affiliations:** 1 Süleyman Demirel University Faculty of Medicine, Department of Pediatric Endocrinology Endocrinology and Diabetes, Isparta, Turkey; 2 Konya Research Hospital, Department of Pediatrics Endocrinology and Diabetes, Konya, Turkey; 3 GATA Medical Faculty, Department of Pediatrics, Ankara, Turkey; 4 Konya Research Hospital, Department of Biochemistry, Konya, Turkey; 5 Katip Çelebi University Faculty of Medicine, Department of Pediatric Endocrinology, İzmir, Turkey

**Keywords:** Non-alcoholic fatty liver disease, total antioxidant status, oxidative stress, obesity, insulin resistance, childhood

## Abstract

**Objective:** Non-alcoholic fatty liver disease (NAFLD) has become one of the most common chronic liver diseases in children. The aim of this study was to investigate the associations of oxidative stress with insulin resistance and metabolic risk factors in obese adolescents with NAFLD.

**Methods:** Forty-six obese adolescents (23 girls and 23 boys, mean age: 12.8±2.2 years) and 29 control subjects (15 girls and 14 boys, mean age: 12.7±2.7 years) were enrolled in the study. The obese subjects were divided into two groups (NAFLD group and non-NAFLD group) based on the elevated alanine aminotransferase levels (>30 IU/L) and the presence or absence of liver steatosis detected by ultrasonography. Insulin resistance was evaluated by homeostasis model assessment (HOMA-IR) from fasting samples. Plasma total antioxidant status (TAS) and total oxidant status (TOS) level measurements (REL Assay Diagnostics) were done in all participants. The ratio of TOS to TAS was regarded as an oxidative stress index (OSI), an indicator of the degree of OS.

**Results:** Fasting insulin levels and HOMA-IR values in the NAFLD group were significantly higher than in the non-NAFLD and control groups. TAS measurements were decreased in both obese groups (NAFLD and non-NAFLD) in comparison with the control group. TOS and OSI measurements were higher in the NAFLD group than in the non-NAFLD and control groups. OSI was positively correlated with fasting insulin (r=0.67, p=0.01) and HOMA-IR (r=0.71, p=0.02) in the NAFLD obese group.

**Conclusions:** In this cross-sectional study, elevated OS markers in obese adolescents with NAFLD were associated with insulin resistance. This data suggest that an antioxidant therapy might have a potential for treating NAFLD associated with insulin resistance.

**Conflict of interest:**None declared.

## INTRODUCTION

The prevalence of obesity has increased dramatically in children and adolescents in both developed and developing countries and is becoming an important medical problem. In recent years, pediatric non-alcoholic fatty liver disease (NAFLD) has increased in line with the increased prevalence of pediatric obesity and has also become an important worldwide health problem (1,2). The estimated prevalence of pediatric NAFLD varies between 2.6% and 9.6% and is associated with sex, age, and ethnicity (3,4).

Fatty liver, the earliest and most prevalent stage of NAFLD (5,6), is thought to sensitize the liver to additional necroinflammatory insults (7), thus promoting disease progression to steatohepatitis and cirrhosis. A number of factors point to the multifactorial nature of this disease, including derangement in metabolic parameters, endotoxin-induced cytokine release, and oxidative stress (OS). Several lines of evidence suggest that chronic OS may be important in the progression of NAFLD (8). OS can result from increased reactive oxygen species (ROS) or reactive nitrogen species (RNS) production or from decreased anti-oxidants. ROS or RNS from mitochondria, endoplasmic reticulum, and peroxisomes play an important role in the pathogenesis of NAFLD. Hepatic injury develops as hepatocytes and their anti-oxidant defenses are overwhelmed by OS as appears to be the case in NAFLD (9).

In adult studies, NAFLD was found to be associated with an increase in serological parameters of OS. Loguercio et al (10) detected the disorder in redox status in more than 90% of the patients, evidenced by significant enhancement in malondialdehyde (MDA; 92%) and 4-hydroxynonenal (98%) levels in erythrocytes from NAFLD patients and a reduction in antioxidant capacity of plasma, in comparison with values in healthy subjects. Furthermore, Videla et al (11) found that progressive functional deficiency associated with the development of liver injury contributed to the lower activity of antioxidant enzymes in adult patients with NAFLD. Koruk et al (12) found that serum MDA and nitric oxide (NO) levels were significantly increased in adult patients with non-alcoholic steatohepatitis (NASH), indicating increased OS, but they found no correlations between serum MDA and NO levels and the histopathologic findings in NASH patients.

Several studies have suggested that the plasma total antioxidant status (TAS) and total oxidant status (TOS) parameters may be useful non-invasive markers of OS-related diseases such as multiple sclerosis, chronic hepatitis, coronary artery disease, hepatitis B infection, intrauterine growth restriction, and diabetic neuropathy. Therefore, the ratio of TAS to TOS, expressed as OS index (OSI), has proven to be helpful in determining the net OS. Currently, information on associations between OS status and NAFLD is lacking in children. Therefore, we investigated serum concentrations of OS markers in obese adolescents with NAFLD. Clarification of these relationships may help to suggest the possible underlying mechanisms and may be of clinical importance in planning preventive and therapeutic strategies.

## METHODS

**Study Population**

Forty-six obese adolescents (23 girls and 23 boys, mean age: 12.8 ± 2.2 years, age range 12-17 years, mean body mass index (BMI): 30.82±1.29) were randomly recruited from obese children who were admitted to the Pediatric Endocrinology Unit of Konya Research Hospital from January 2010 to April 2011. The obese subjects was divided into two groups: (1) NAFLD group (11 girls and 12 boys, mean age: 13.1±2.1 years, mean BMI: 31.02±1.14) with high alanine aminotransferase (ALT) levels (ALT >30 IU/L) and ultrasound evidence of fatty changes in the liver and (2) non-NAFLD group (12 girls and 11 boys, mean age: 12.5±2.2 years, mean BMI: 30.63±1.49) with low ALT levels (ALT <30 IU/L) and with no ultrasound evidence of fatty changes in the liver). Lean adolescents (15 girls and 14 boys, mean age: 12.7±2.7 years with a mean BMI of 18.36±3.97) were also enrolled in the study as a control group. This control group consisted of healthy adolescents who attended the hospital for minor illnesses such as common cold, conjunctivitis, or other similar conditions. None of the subjects were vegetarian.

Exclusion criteria were hepatic virus infections (hepatitis A, B, C, cytomegalovirus and Epstein-Barr virus infections), alcohol consumption, history of parenteral nutrition, and use of drugs known to induce steatosis (e.g. amiodarone, glucocorticoids, L-asparaginase, valproic acid) or to affect body weight and carbohydrate metabolism. Autoimmune and metabolic liver disease, Wilson’s disease, and α-1-antitrypsin-associated liver disease were ruled out using standard clinical and laboratory criteria. Family history for obesity and diabetes were obtained by questionnaires. None of the patients had a family history for type 2 diabetes.

The study protocols were approved by the institutional review board of Konya Training and Research Hospital (approval number 201119). Informed consent forms were signed by the parents of the children.

**Anthropometric Measurements**

Height was measured to the nearest 0.5 cm, without shoes, back against the wall, eyes looking straight ahead, with a right-angled triangle resting on the scalp and against the wall. Weight measurements were done with the subjects in the postabsorptive state, with an empty bladder, without shoes and in light undergarments, using a lever scale, sensitive to 100 g, BMI was calculated as weight (in kilograms) divided by height (in meters squared). Patients with a BMI of ≥95th percentile according to reference curves for Turkish children and adolescents were accepted as obese (13).

Pubertal development stages were assessed by the same pediatric endocrinologist using the criteria of Tanner stages. Staging for sexual maturation was greater than 2 in all patients and controls (Tanner stages II-IV).

**Blood Pressure**

After the child had rested for at least 5 min and was in a sitting position, diastolic and systolic pressure (mmHg) measurements were taken, using a mercury-gravity manometer and a cuff appropriate for body size.

**Blood Samples and Insulin Sensitivity Markers**

Serum glucose, insulin levels and other parameters were determined in blood samples collected between 08.00 and 10.00 am, after fasting for 12h overnight. Glucose was determined by the glucose oxidase method. Serum insulin levels were measured by an Immulite immunoassay system (Diagnostic Products, Los Angeles, CA). Serum concentrations of total cholesterol, high-density lipoprotein cholesterol (HDL-cholesterol), and triglycerides were measured using routine enzymatic methods with Olympus 2700 analyzer (Olympus Diagnostica GmbH, Hamburg, Germany). Low-density lipoprotein cholesterol (LDL-cholesterol) levels were calculated using Friedewald’s equation. Standard liver function tests [ALT, aspartate aminotransferase (AST)] were measured on the same day with an autoanalyzer.

Insulin sensitivity index was derived from fasting blood samples. The homeostasis model assessment of insulin resistance (HOMA-IR) was calculated as fasting insulin concentration (μU/mL) ? fasting glucose concentration (mmol/L)/22.5.

**TOS Determination**

Serum samples for the measurement of TAS and TOS were stored at -80°C until needed. Serum TOS was measured using Erel’s TOS method (14) (REL Assay Diagnostics), which is based on the oxidation of ferrous ion to ferric ion in the presence of various oxidative species in the acidic medium. Ferric ion was measured by xylenol orange. The test parameters were as follows: method, end-point measurement; serum volume, 10 μL; R1 volume, 200 μL; R2 volume, 50 μL; reaction time, 10 min; temperature, 37°C; read points, 34; primary wave length, 560 nm; and secondary wave length, 800 nm. The results were expressed in μmoL H2O2 equivalent/L (μmol H2O2 eq/L).

**TAS Determination**

TAS was measured colorimetrically using the Total Antioxidant Status kit (15) (REL Assay Diagnostics). The color change was measured using Olympus 2700 analyzer. The reaction was calibrated with Trolox (a water-soluble analogue of vitamin E, 6-hydroxy-2.5.7.8-tetramethylchroman-2-carboxylic acid), and the TAS value of the samples tested was expressed as μmol Trolox equivalent/L (μmol Trolox eq/L).

**Oxidative Stress Index**

OSI was defined as TOS to TAS ratio (16,17,18) and was calculated as follows: OSI (arbitrary unit) = ((TOS, μmol H2O2 eq/L)/(TAS, μmol Trolox eq/L)).

**Liver Ultrasonography**

All patients with abnormally high transaminase levels and those with abnormal liver ultrasound images were screened for other liver conditions (hepatitis B surface antigen, hepatitis C antibody, prothrombin time, serum iron level, total iron-binding capacity, ferritin, and antinuclear antibodies) which were all negative. Liver ultrasonography was performed by a trained operator who was blinded to all clinical and laboratory characteristics of the participants. Scans were performed in all subjects using a General Electric Logic 9 (MI, USA) machine, equipped with 7.5 MHz probes in younger children and 5 MHz probes in larger or markedly obese children and adolescents. The presence of NAFLD was assessed by the scoring system defined by Tominaga et al (4) according to the hyperechogenicity of liver tissue, difference in echogenicity between liver and diaphragm, and visibility of vascular structures. The diagnosis of NAFLD is usually made from mild elevations in liver enzymes during a routine blood testing and liver ultrasonography in an obese child. Although liver ultrasonography cannot determine whether fibrosis or inflammation is present, it has a sensitivity of 89% and a specificity of 93% for detecting histological steatosis (19). Elevated ALT was defined as >30 IU/L in our study (20,21). In the present study, all obese patients with NAFLD had high ALT levels.

**Statistical Analysis**

Mean and standard deviations (SD) were used as descriptive statistics. Differences in the means of variables were tested using both parametric and nonparametric tests depending on the distribution of the variables. The correlations of OSI with BMI-SD score (SDS), systolic-diastolic blood pressure, lipids, fasting glucose-insulin, transaminases, and HOMA-IR were analyzed using the Pearson correlation coefficient (r). Spearman’s rank correlation coefficient was applied to analyze the associations between categorical variables (TAS, TOS, OSI). Comparison between groups was performed using ANOVA (post hoc: Bonferroni correction). A probability value of <0.05 was considered significant. The SPSS version 14 (SPSS, Chicago, IL, USA) was used for statistical analyses.

## RESULTS

Table 1 shows the anthropometric, physical, and biochemical parameters in the obese and lean groups. There was no significant difference between the non-NAFLD and NAFLD obese groups for BMI-SDS (p=0.12). These groups also showed no significant differences in terms of diastolic blood pressure. However, both obese groups had significantly higher systolic blood pressure values than the control group.

HDL-cholesterol levels were higher in the non-NAFLD group than in the NAFLD group (p=0.03). However, the control group had significantly higher HDL-cholesterol levels compared to both NAFLD and non-NAFLD groups. Also, no significant difference was found between NAFLD and non-NAFLD groups for other lipid levels. However, in the NAFLD group, triglyceride levels were found higher than in the lean group (p=0.04). Both the non-NAFLD and lean groups showed no significant differences in terms of triglycerides, total cholesterol, and LDL-cholesterol levels.

The mean fasting serum glucose levels of the groups were similar at admission. The non-NAFLD group had significantly higher fasting insulin levels than the lean group (p=0.01). However, the NAFLD group had significantly elevated fasting insulin levels compared to both control and non-NAFLD groups (p<0.001). HOMA-IR values were found to be higher in the non-NAFLD group than in the healthy lean group (p=0.04), and the NAFLD group had significantly greater HOMA-IR values compared with the non-NAFLD group (p=0.02) and also with the control group (p=0.01). ALT levels were significantly higher in NAFLD group compared to non-NAFLD and control groups. However, there was no significant difference between all groups for AST levels.

The NAFLD group had significantly higher levels of TOS and OSI than the non-NAFLD (p=0.03) and control groups (p=0.01) (Figure 1a, 1c). TAS measurements were decreased in both obese groups (NAFLD and non-NAFLD) in comparison with the control group. However, the NAFLD group had significantly lower measurements of TAS than the non-NAFLD (p=0.04) and control groups (p=0.02) (Figure 1bt).

OSI was positively correlated with fasting insulin level (r=0.67, p=0.01) and HOMA-IR values (r=0.71, p=0.02) in the NAFLD group. OSI was negatively correlated with TAS in the non-NAFLD group (r=-0.76, p=0.001) and in the NAFLD group (r=-0.57, p=0.004). Serum OSI levels were not significantly associated with total cholesterol, HDL-cholesterol, LDL-cholesterol, ALT or AST levels (Table 2).

## DISCUSSION

Several studies have implicated OS in adult NAFLD (22,23). However, very little is known about the involvement of oxidative damage in paediatric obesity-related fatty liver (24). The natural history and pathogenesis of NAFLD are poorly understood in children. Clearly, NAFLD is associated with obesity in children; however, only a subset of obese children develops NAFLD. Insulin resistance is generally accepted to be an essential pathophysiological factor or ‘‘first hit’’ in the development of NAFLD (4). Alterations in hepatic fat metabolism result in the accumulation of lipid droplets within the hepatocytes (25). To progress from steatosis to injury leading to inflammation and fibrosis, it has been hypothesized that OS or a ‘‘second hit’’ is required (7). The source of increased OS may be diet, environment, infections, drugs, or toxins. Alternatively, increased OS may be due to a relative deficiency of dietary or endogenous antioxidants (25). In order to evaluate the impact of NAFLD on oxidative status, we measured TAS, TOS levels, and OSI. To our knowledge, there is no study reported in the literature related to TAS, TOS levels, and OSI in obese children with NAFLD. Before effective treatment regimens can be designed for children, susceptibility to and mechanisms underlying NAFLD progression must be elucidated. Levels of many oxidant and antioxidant parameters present in serum may be measured individually. Since oxidant and antioxidant parameters show an additive effect, individual values may not correctly reflect TOS or TAS (11,12). Therefore, TAS and TOS are more accurate indicators of oxidative and antioxidative status of individuals. In this study, we have used novel measurement methods to evaluate the extent of OS in obesity with NAFLD in children.The alteration of the antioxidant mechanisms in obesity has been shown in both humans and in experimental animal models (26). Chrysohoou et al (27) have reported that obesity and, especially, central adiposity are correlated with decreased antioxidant capacity, irrespective of age, metabolic and various lifestyle variables in adults. Both Olusi et al (28) and Ozata et al (29) found that superoxide dismutase and glutathione peroxidase activities were lower in obese persons as compared with non-obese persons. Melissas et al (30) have found that plasma antioxidant capacity is impaired in morbidly obese adult patients. One of the most recent studies by Furukawa et al (31) demonstrated that plasma adiponectin concentrations correlated inversely with systemic OS and that probably increased oxidative stress in accumulated fat leads to dysregulated production of adipocytokines. Nobili et al (32) found that a high proportion (83%) of children with NAFLD show signs of oxidative injury, as evaluated by elevated circulating levels of protein carbonyls. Molnar et al (33) demonstrated that the lipid corrected values of the most important fat-soluble antioxidant vitamins, i.e. α-tocopherol, α and β-carotene, as well as the plasma TAS concentrations were significantly lower in obese children with metabolic syndrome than in those without metabolic syndrome even after adjustment for fat mass and age. In a study by Fierbinteanu-Braticevici et al (34), serum OSI has been suggested as an independent risk factor for fibrosis in the course of NASH.

Our study showed that obese adolescents with NAFLD had increased OSI values, a finding which was positively correlated with an insulin resistance. TOS levels and OSI values were higher in the NAFLD group than in the non-NAFLD and lean groups. We emphasized that having NAFLD in obese adolescents has a harmful effect on TAS. We found that the oxidative/antioxidative balance shifted toward the oxidative side, and that a state called increased oxidative stress was present in obesity with NAFLD. In a few studies on NASH subjects, antioxidant and oxidant capacities have been investigated (11,12,34). Koruk et al (12) reported an increase in OS in NASH subjects. However, in their study, plasma anitoxidant capacities were observed to be comparable in NASH subjects and controls. These authors suggested that impaired antioxidant defense mechanisms in responding to increased OS might be an important factor in the pathogenesis of NASH.

Horoz et al (18) found in their adult study that TAS levels were 0.85±0.11 and 1.88±0.32 mmol Trolox eq/L in subjects with NASH and controls, respectively (p<0.05). OSI was reported to be 64±14 and 19±11 in subjects with NASH and controls, respectively (p<0.05). We found that TAS levels were 0.58±0.06 and 0.82±0.12 mmol Trolox eq/L in pediatric subjects with NAFLD and controls, respectively, and that OSI levels were 16.32±2.16 for adolescent NAFLD patients and 7.36±2.14 for lean controls. The adolescent NAFLD patients had lower TAS and OSI measurements than adults.

Fasting serum insulin and HOMA-IR were significantly higher in the overweight children (35), reaffirming the essential pathophysiological role of insulin resistance in the development of NAFLD. Insulin resistance leads to increased lipolysis and free fatty acid output. The influx of free fatty acid into the liver, combined with alterations of the fat metabolism in the liver, results in the accumulation of triglycerides within the hepatocytes (36,37). Lipid-laden hepatocytes are believed to be susceptible to a second-hit injury by compounds, including oxygen radicals and endotoxins (36). Insulin resistance is involved in the development of not only steatosis but also fibrosis by increasing fatty acid β-oxidation and oxidative stress (38). Insulin resistance will predispose to disturbances in lipid metabolism, including elevated triglycerides, as observed in our study. In the present study, we found higher fasting insulin levels in obese adolescents with NAFLD compared to the other groups. In our study, the NAFLD group had significantly greater HOMA-IR values compared with the non-NAFLD group and also with the control group. Our study showed that OSI was positively correlated with insulin and HOMA-IR in the NAFLD group. Ko et al (39) reported that 96% of their pediatric patients with NAFLD demonstrated insulin resistance, which was defined as HOMA-IR>2. Radetti et al (40) showed a decreased insulin sensitivity in all of the obese children, but no difference was found in insulin sensitivity between children with or without NAFLD. Chan et al (41) showed a positive correlation between insulin resistance markers and presumed NASH only in male obese children.

Screening for chronic liver disease is most commonly done using serum ALT activity. Pediatric clinical trials use ALT to exclude potential subjects with liver disease. Clinical trials commonly use a cutoff of 2 to 3 times the upper limit of normal as a means to exclude subjects with liver disease. Among all National Health and Nutrition Examination Survey participants aged 12 to 17 years, before excluding those with risk factors for liver disease, the 95th percentile cutoff value for ALT was 37.2 U/L in boys and 26.0 U/L in girls. Schwimmer et al (21) reported that the 95th percentile for ALT in their study was 25.8 U/L in boys and 22.1 U/L in girls. The threshold of ALT >25 U/L for boys and >22 U/L for girls is consistent with the prevalence of pediatric fatty liver disease as estimated by a population-based autopsy study. In the present study, all obese patients with NAFLD had high ALT levels defined as >30 IU/L.

The diagnosis of NAFLD is usually made by mild elevations in liver tests during a routine blood testing and liver ultrasonography in an overweight or obese child. Although liver ultrasonography can estimate neither fibrosis nor inflammation, it has a sensitivity of 89% and a specificity of 93% for detecting histological steatosis (19). The most important limiting factor of our study was that the stage of NAFLD was not determined by liver biopsy. There is no option except biopsy for determining the spectrum of disease in a patient with liver steatosis determined by ultrasonography and mildly elevated liver enzymes. However, liver biopsy was not performed in patients with NAFLD in our study as there is no proven therapy based on biopsy findings and because of its related cost and risk.

In conclusion, in this study obese adolescents with NAFLD were shown to have higher TOS and lower TAS values than lean control subjects. Also, there was a significant relationship between OSI and insulin sensitivity in obese adolescents with fatty liver. Further studies with a higher power to determine the cause and effect relationship between fatty liver and oxidation in obese adolescents are warranted.

## Figures and Tables

**Table 1:**
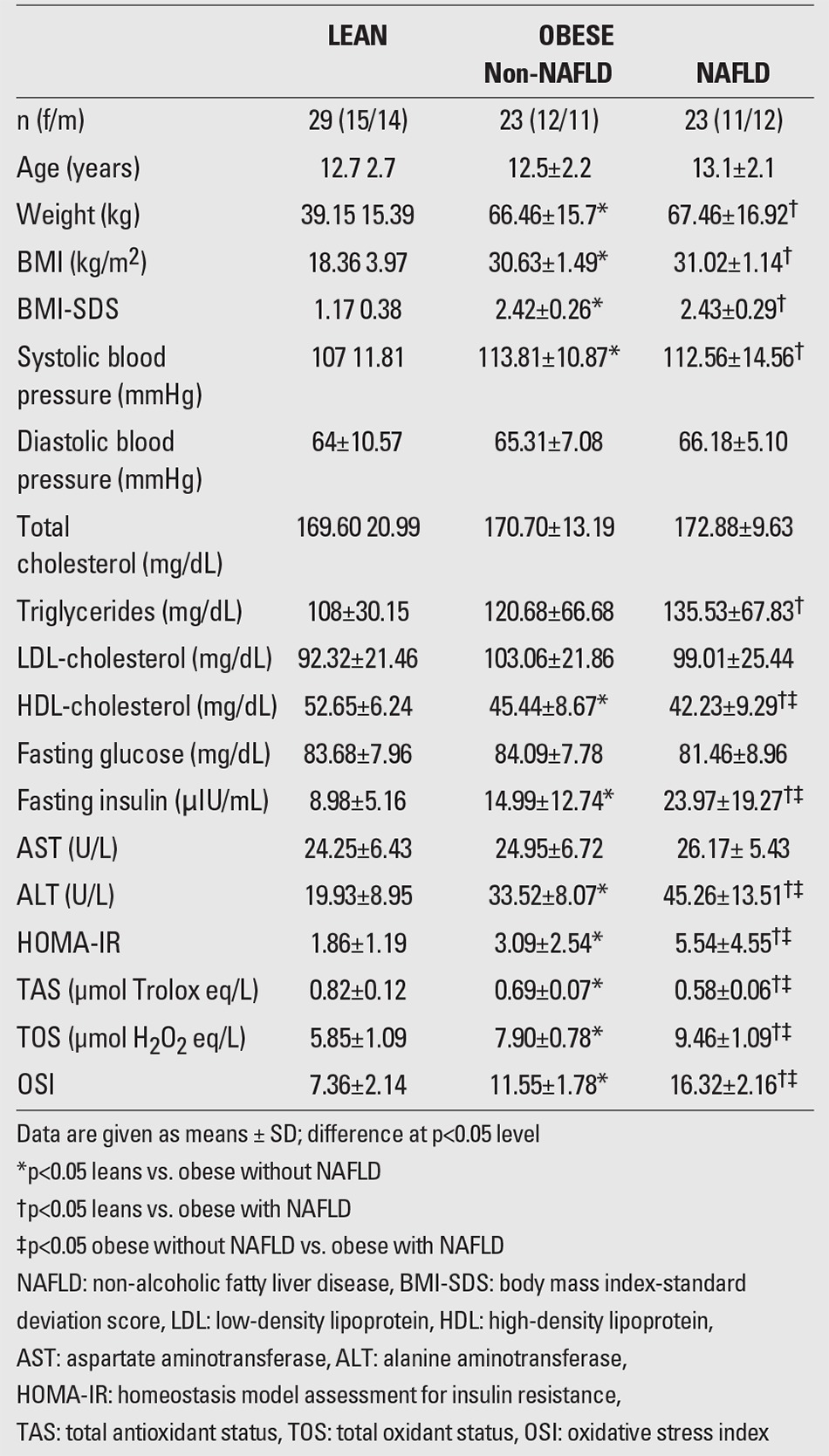
Characteristics of lean and obese children

**Table 2:**
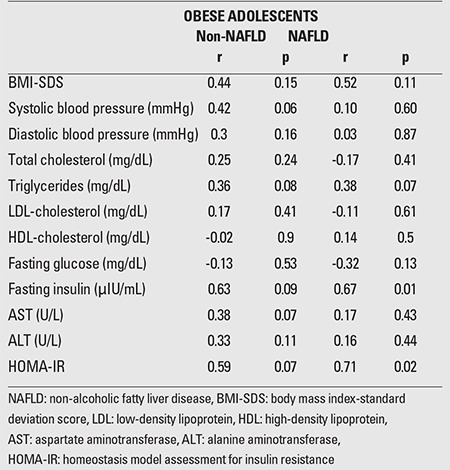
Pearson correlations between oxidative stress index (OSI) and metabolic parameters in obese children with NAFLD and non-NAFLD groups

**Figure 1a:**
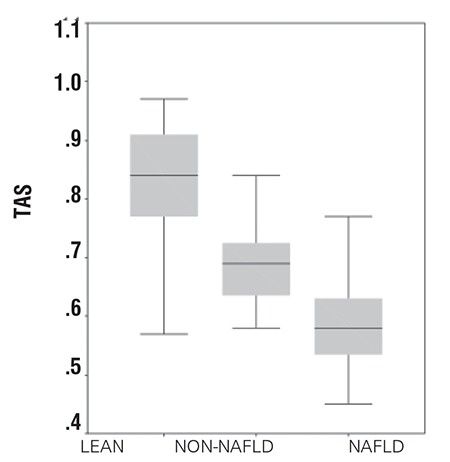
Comparison of total antioxidant status (TAS) in groups

**Figure 1b:**
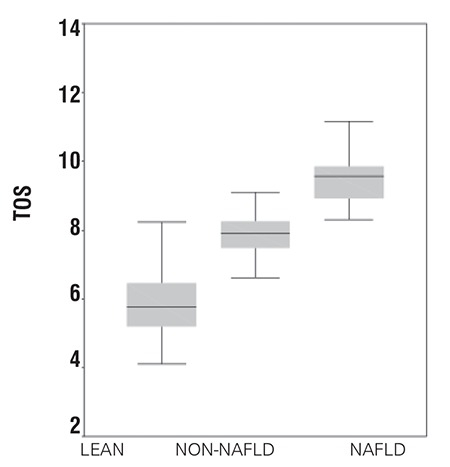
Comparison of total oxidant status (TOS) in groups

**Figure 1c:**
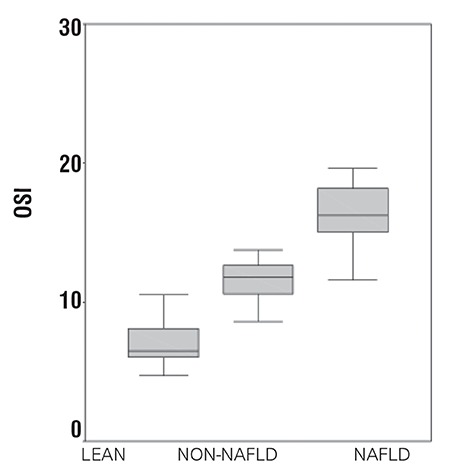
Comparison of oxidative stress index (OSI) in groups
